# Novel use of three administrative datasets to establish a cohort for environmental health research

**DOI:** 10.1186/s12889-015-1580-1

**Published:** 2015-03-14

**Authors:** Lucy F Telfar Barnard, Michael G Baker, Simon Hales, Philippa Howden-Chapman

**Affiliations:** He Kainga Oranga/Housing and Health Research Programme, University of Otago, Newtown, Wellington, 6021 New Zealand

## Abstract

**Background:**

In recent years publications have called for increased use of administrative data for research; predicted that use would rise; and discussed possible ethical parameters for that use. This paper describes the novel combination of three administrative datasets to create a population cohort for environmental health research, and investigates the potential use of a national health register as a total population denominator.

**Methods:**

We matched a national health register (the New Zealand national health index or NHI) to Quotable Value New Zealand Ltd (QV) nationwide residential dwelling data, and to hospital admissions data, to create a national matched cohort with health outcomes for the period 2000 – 2006. We then compared population distribution and hospitalisation rates by gender, age, ethnic group and Census Area Unit-based socio-economic deprivation index across the Census, NHI and matched cohort populations.

**Results:**

The NHI population was 23% larger than the Census. Differences between the NHI and Census were most marked in those aged over 90 years; with ethnicity unknown or an unassigned Census area unit; and in Asian Peoples aged under 30 years. The match rate between QV and NHI data was 70%. There were further differences between the NHI and matched cohort populations, particularly for rural areas and older age groups. Compared to Census-based rates, NHI and cohort-based hospitalisation rates were higher in those aged 75 and over, differed by ethnicity, and had less socio-economic gradient.

**Conclusions:**

The NHI was larger than the Census due to record duplication and entries for people residing overseas remaining on file under New Zealand addresses. NHI and QV matching was incomplete due to NHI address data being poor quality or not suitable for matching. To better approximate true hospitalisation rates, studies using the NHI as a cohort should exclude those aged over 90 years; or with ethnic group or Census area unit unknown. Cohort hospitalisation rates should also be adjusted for differences from the Census, particularly the lower hospitalisation rates for those aged 75 and over, and other differences by age, ethnic group and socio-economic deprivation.

## Background

In recent years publications have called for increased use of administrative data for health research [1]; predicted that use would rise [2], and discussed possible ethical parameters for that use [3,4]. There is a growing literature on the use of health-related data sources for public health research [5-8], sometimes linked with other types of data sources [9,10]. However, experiences in combining non-health datasets with health data at a national level are still uncommon. This paper describes the novel combination of a national health register with a nationwide housing dataset, and national public hospitalisation data; to provide a matched national cohort for environmental epidemiological research.

The New Zealand Ministry of Health maintains a register, the National Health Index (NHI), of anyone who has come into contact with the New Zealand public health system since 1988.

The Ministry of Health also collects comprehensive records of all public hospital admissions, known as the National Minimum (Hospitalisations) Dataset (NMDS). Both the NHI and NMDS are regularly used for public health research, and have robust protocols in place to ensure anonymity and ethical use. Use of the New Zealand Census as the denominator for hospital admission rates is so general [11-14] that in most cases it is implicit rather than specified [15].

QV, an independent company owned by the New Zealand Government, maintains a national property database, recording information on dwellings, including construction age, materials and style. Access to QV data is available on commercial terms.

These three datasets were combined to create a cohort for housing and health research (the ‘matched cohort’) with individual-level data on demographic characteristics and housing exposures. NMDS data were linked to the NHI data to assess health outcomes.

New Zealand Census data have previously been treated as a cohort for health research, using probabilistic matching to hospitalisation data. Public health researchers using New Zealand’s Census cohort have indicated that “The reliability of using Health datasets only, with the NHI as a pseudo-population register, for monitoring … into the future requires active consideration and feasibility research…” [16].

This paper assesses the performance of both the NHI population and the matched cohort for use in epidemiology, by comparing their demographic distributions and hospitalisation rates with those of the 2006 Census. The matched cohort has been used for a study of excess winter hospitalisation and housing [17], and the method has been further adapted for use in evaluating the health outcomes of a government insulation subsidy scheme [18], and of mechanised home ventilation systems (in progress).

## Methods

### Data sources and matching

New Zealand’s Ministry of Health provided the authors with addresses and a unique identifier for each NHI record. Before providing addresses to QV, we filtered them to exclude non-New Zealand residents, addresses outside New Zealand, and records for people who had died before the start of the study period, 1 January 2000. Although matches were provided for the population from 1 January 2000, we report here results for the population on the date of the closest New Zealand Census to data provision, 7 March 2006.

QV matched the remaining addresses to their property database, using their own proprietary probabilistic matching system, then provided the matched housing data and unique identifiers to the authors.

**Figure 1:**
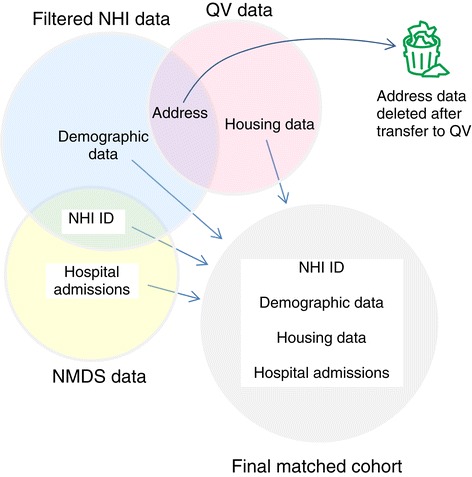
**Data matching.**

The Ministry of Health also provided the authors with demographic data for all NHI records, with unique identifier, as well as hospitalisation data for the period 2000–2006 inclusive. The hospitalisation data were filtered according to a previously-described protocol to account for changes in health-care practices by region and over time [13], including only acute overnight hospitalisations. We linked the housing data with the demographic, and hospitalisation data, to create the matched cohort (Figure 1).

### Data fields

New Zealand’s Ministry of Health maintains a National Health Index (NHI) of anyone who has had contact with the New Zealand health system since 1988. The system was phased in from 1980, starting with the larger regions, and completed by 1988. The database was migrated and centralised in 1993. At the time of this study, the Ministry of Health estimated that 98% of New Zealanders appeared in the NHI database.^a^

NHI data routinely available for health research include the following fields:

- date of birth;
- date of death (if any);
- gender (male, female, unknown);
- ethnicity (as reported, up to three ethnicities stored);
- domicile code, which can be directly mapped to a Census Area Unit (CAU)^b^, from which can be derived indicators of rurality and socio-economic deprivation decile index (NZDep2006) [19]; and
- date of last update.

NHI address data may also be accessed, but with additional limits to protect individual privacy and confidentiality of health information.

Census data was available for this study disaggregated by:

- five-year age group;
- sex (male, female);
- ethnicity (modified total ethnicity); and
- NZDep2006.

In order to compare NHI data to Census data, we converted the three reported ethnicities in the NHI into ‘modified total ethnicity’. The ‘modified total ethnicity’ variable has categories ‘Māori’, ‘Pacific’, ‘Asian’, and ‘non-Māori, non-Pacific, non-Asian’ (non-MPA). Each subject may be categorised in more than one of the first three categories. The residual non-MPA comparator group is predominantly of European ethnicity.

### Population and hospitalisation comparisons

We filtered the total NHI population to exclude people who were not New Zealand residents, who had addresses outside New Zealand, who were aged over 100 years on the study Census date, or whose record had not been updated since NHI data were centralised in 1993. We then compared the 7 March 2006 New Zealand resident NHI population with the 7 March 2006 New Zealand Census by gender, age group, ethnic group, and NZDep2006 quintile. We performed a multivariate poisson regression to identify demographic categories in which NHI records appeared to include most duplicates or unreliable data.

After comparing NHI and Census populations, we excluded as likely duplicates NHI records with unknown ethnicity, unassigned socio-economic index NZDep2006, or aged over 90 years, from the matched cohort and from subsequent analysis.

We next measured QV and NHI data match rates, using poisson regression to measure the remaining NHI distribution with the matched cohort distribution by gender, age, ethnic group, NZDep2006 quintile and rurality category, to identify differences in match rates by variable. Last, we calculated and compared hospitalisation rates for the six years 2000 – 2005 inclusive, across the Census, NHI and matched cohort populations. As all three populations were so large, even small size differences between them were statistically significant, so decisions on which differences were meaningful could only be made subjectively.

### Preserving health record anonymity

Combining hospitalisation data with environmental data by address could risk the anonymity of health information. We preserved anonymity for the matched cohort by ensuring that the company carrying out the data matching (QV) did not have access to the hospitalisation records, and that we did not retain address records once they had been provided to QV. The address-matched environmental data were back-matched to hospitalisation data using the unique identifier number assigned by the Ministry of Health. This unique identifier number was specific to this study, and not the standard encrypted NHI number regularly provided with Ministry of Health data, so that there was no possibility address data could be combined with routinely released hospitalisation data, or be used in any other context.

We gained ethical approval from the New Zealand Health and Disability Multi-Region Ethics Committee prior to embarking upon this research (MEC/06/09/106).

## Results

### NHI vs 2006 Census population comparisons

#### Preliminary exclusions

**Figure 2:**
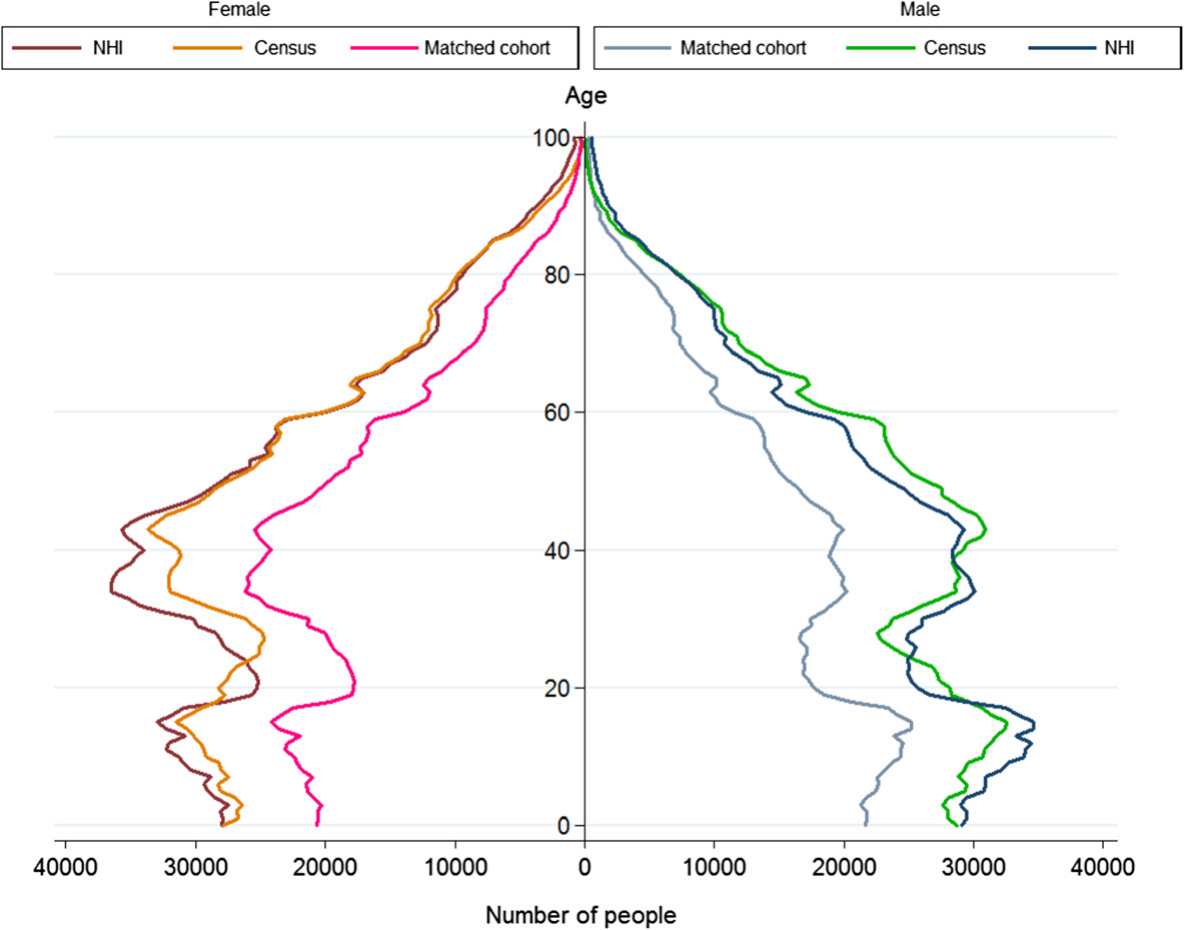
**NZ male and female populations: NHI cohort, Census, and matched cohort by age, 2006.** *Legend*: 7 March 2006 living NZ residents with known NZDep quintile and known ethnicity only. Only NHI and matched cohort records updated since 1993.

The New Zealand Ministry of Health provided 6,518,508 NHI records. Of these, 855,914 were excluded as not yet born, or deceased before, 7 March 2006. A further 720,462 were excluded as aged over 100 years, being non-New Zealand residents, having an address outside New Zealand, or having a record not updated since the NHI was centralised in 1993. The remaining 7 March 2006 New Zealand resident NHI population (4,942,132) was 23% larger than the comparable 7 March 2006 Census population (4,027,941) (Figure 2 and Table 1).

**Table 1:** **NHI and Census populations by demographic sub-groups, 2006**

	**NZ resident population 7 March 2006**		**NHIcensus**	**NHI overcount rate ratio**	
	**NHI**	**Census**		**Poisson regression all variables, 95% CI**	
**Total**	4,942,132	4,027,941	1.23		
**Sex**					
Male	2,404,629	1,965,597	1.22	Reference	
Female	2,537,503	2,062,344	1.23	0.01	(0.01-0.02)
**Age group**					
0-4 yrs	308,947	275,079	1.12	Reference	
5-14 yrs	727,676	592,497	1.23	0.03	(0.03-0.03)
15-24 yrs	687,321	571,176	1.20	0.00	(0.00-0.01)
25-34 yrs	713,075	519,015	1.37	0.05	(0.05-0.06)
35-44 yrs	789,418	615,258	1.28	0.03	(0.03-0.04)
45-54 yrs	651,461	546,138	1.19	0.00	(0.00-0.01)
55-64 yrs	474,201	413,175	1.15	−0.02	(−0.02--0.01)
65-74 yrs	300,119	265,491	1.13	−0.03	(−0.04--0.03)
75-84 yrs	199,783	173,448	1.15	−0.04	(−0.04--0.03)
85-89 yrs	50,314	38,124	1.32	0.01	(0.00-0.02)
90+ yrs	39,817	18,540	2.15	0.17	(0.16-0.18)
**Ethnic group**					
Non-MPA	3,197,120	2,727,771	1.17	Reference	
NZ Māori	559,112	565,335	0.99	−0.15	(−0.16--0.15)
Pacific Peoples	312,441	265,965	1.17	−0.10	(−0.11--0.10)
Asian Peoples	299,147	354,573	0.84	−0.19	(−0.19--0.18)
Unknown	599,254	167,799	3.57	0.33	(0.33-0.34)
**NZDep2006 quintile**					
1-2	724,315	825,606	0.88	Reference	
3-4	795,048	810,843	0.98	0.06	(0.06-0.06)
5-6	901,914	797,037	1.13	0.13	(0.13-0.14)
7-8	1,074,515	791,394	1.36	0.22	(0.22-0.22)
9-10	1,178,584	798,162	1.48	0.27	(0.27-0.27)
Unknown	267,756	4,899	54.66	0.74	(0.73-0.74)

#### Age and sex distribution

The NHI overcount for most age groups ranged from 12% to 37% (Table 1). However, the overcount was 115% for those aged 90+ years. Consequently, results for this age group were excluded from the matched cohort and subsequent calculations for both populations, including hospitalisation rates. The Census population dipped among those aged in their late 20s. In the NHI data, this trough was centred earlier, at 22 rather than 27 years (Figure 2).

#### Distribution by ethnic group

The population with ethnic group unknown was 2.57 times higher in the NHI than in the Census, and was therefore excluded from subsequent calculations as likely duplicates (Table 1). The NZ Māori population was similar across the two populations, but Asian peoples were under-represented in the NHI compared to the Census, and Pacific and non-MPA were over-represented.

#### Age and ethnic group distribution

**Figure 3:**
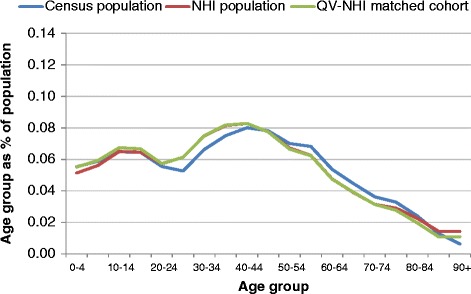
**Age group distribution: Census, NHI, and matched cohort - Non-Māori, non-Pacific, non-Asian, 7 March 2006.**

**Figure 4:**
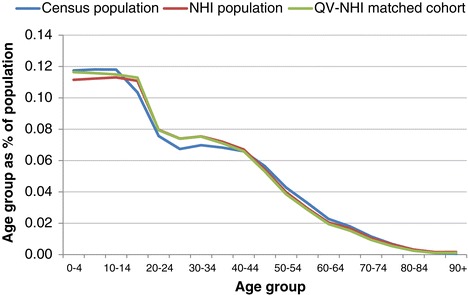
**Age group distribution: Census, NHI, and matched cohort - Māori, 7 March 2006.**

**Figure 5:**
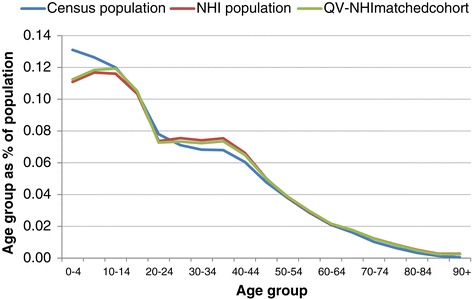
**Age group distribution: Census, NHI, and matched cohort by age group - Pacific Peoples, 7 March 2006.**

**Figure 6:**
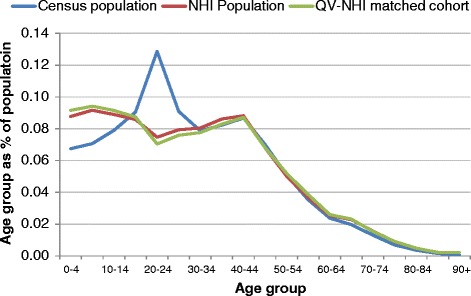
**Age group distribution: Census, NHI, and matched cohort by age group - Asian Peoples, 7 March 2006.**

Figures 3, 4, 5 and 6 show age group populations as a percentage of the total population for each ethnic group across the Census, the NHI, and the matched cohort. The Census and NHI populations are broadly similar, except that:

- for non-MPA, the Census population has a higher percentage of people in age-groups between 50 and 84, and lower in age-groups between 25 and 44, and 90 and over (Figure 3);
- for Māori, the Census has a higher percentage of people in age-groups under 15 and between 45 and 79, and lower in age-groups between 15 and 39 (Figure 4);
- for Pacific Peoples, the Census has a markedly higher proportion of the population aged 0–4, and also more 5 to 9 years, but a smaller proportion for those aged 25–49 (Figure 5);
- Asian peoples showed the greatest mismatch in population distributions, with the Census distribution showing much lower percentages of people aged under 15, and much higher percentages for those aged 20–29 (Figure 6).

#### Distribution by socio-economic status

As the NHI population with an unknown or unassigned NZDep2006 quintile was 58 times larger than the Census population with unassigned NZDep2006, we excluded it from all subsequent calculations (Table 1).

#### Distribution by age, ethnicity and socio-economic status

The Census and NHI populations compared across ethnic group and NZDep2006 quintiles showed the population distributions by age group to be roughly similar, the one exception being the Asian peoples’ Census population, which was substantially larger than the NHI population in the 20–24 years age group, across all NZDep2006 quintiles.

### NHI and QV matched data

After excluding NHI records aged 90 years and over and records with unknown ethnicity or NZDep2006 quintile, 4,076,824 records were provided for QV matching. These exclusions also reduced the NHI/Census overcount from 23% to 6%.

#### Match rates

Match rates were 70% nationwide, 74% in “Main Urban” areas and up to 80% in some larger cities. Common reasons for addresses being unmatched were that the address was:

- unsuitable for matching (e.g. Post Office boxes and “private bags”, street number or entire address missing or marked as inaccurate, “rural delivery” addresses with no specific dwelling identified), which made up approximately 2% of all addresses;
- “care of” another address, as these were deemed unlikely to represent the individual’s residential address;
- difficult to confidently identify: for example, a second dwelling at 1 High St may be recorded as 2/1 High St, 1/2 High St, 1A High St, or 1B High St.

#### NHI and Census populations vs matched cohort

The matched cohort (2,841,868) was approximately 26% smaller than the Census (Figure 2 and Table 2). The NHI population and the matched cohort were generally similar in demographic distribution. Notable differences between the NHI population and the matched cohort were in distribution by age, with higher match rates in age groups under 20, and lower match rates, below 60%, in those aged over 80 years; and by ethnicity, with the matched cohort having a higher proportion of Māori and Pacific Peoples than the NHI population.

**Table 2:** **NHI and matched cohort populations by age group, 2006**

	NZ resident population* 7 March 2006		**Match rate**	**NHI overcount rate ratio**	
	**NHI**	**Cohort**	**Cohort NHI**	**Poisson regression all variables, 95% CI**	
**Total**	4,076,824	3,838,119	0.70		
**Sex**					
Male	1,939,949	1,335,200	0.69	Reference	
Female	2,136,875	1,506,668	0.71	0.01	(0.01-0.02)
**Age group**					
0-4 yrs	286,271	211,013	0.74	Reference	
5-14 yrs	634,837	458,886	0.72	−0.01	(−0.01-0.00)
15-24 yrs	559,405	395,137	0.71	−0.03	(−0.03--0.02)
25-34 yrs	578,160	399,507	0.69	−0.04	(−0.05--0.04)
35-44 yrs	642,419	446,121	0.69	−0.03	(−0.04--0.03)
45-54 yrs	527,984	364,903	0.69	−0.03	(−0.04--0.03)
55-64 yrs	387,451	267,238	0.69	−0.03	(−0.04--0.03)
65-74 yrs	249,283	171,477	0.69	−0.04	(−0.04--0.03)
75-84 yrs	168,719	106,199	0.63	−0.10	(−0.11--0.09)
85-89 yrs	42,295	21,387	0.51	−0.24	(−0.26--0.23)
**Ethnic group**					
Non-MPA	2,999,269	2,069,696	0.69	Reference	
NZ Māori	533,291	377,854	0.71	0.01	(0.01-0.02)
Pacific Peoples	287,148	220,139	0.77	0.03	(0.02-0.03)
Asian Peoples	280,431	191,490	0.68	−0.04	(−0.05--0.04)
**NZDep2006 quintile**					
1-2	618,705	441,187	0.71	Reference	
3-4	687,517	455,011	0.66	−0.02	(−0.03--0.02)
5-6	783,175	540,159	0.69	−0.02	(−0.03--0.02)
7-8	946,406	678,209	0.72	−0.02	(−0.02--0.02)
9-10	1,041,021	727,302	0.70	−0.05	(−0.05--0.04)
**Rural classification**					
Main urban	2,988,399	2,203,487	0.74	Reference	
Secondary urban	286,447	193,980	0.68	−0.05	(−0.05--0.04)
Minor urban	376,479	263,203	0.70	−0.03	(−0.03--0.02)
Rural centre	104,015	52,498	0.50	−0.24	(−0.24--0.23)
Other rural	320,973	128,568	0.40	−0.40	(−0.41—0.40)

*New Zealand residents with New Zealand addresses, aged <90 years, with ethnicity and NZDep quintile known.

### Comparison of hospitalisation rates by NHI and 2006 Census denominators

**Figure 7:**
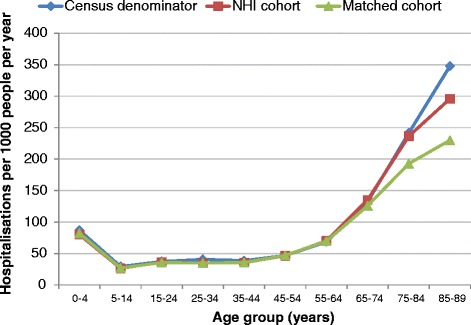
**Hospitalisation rates for 2006 Census, NHI, and matched cohort populations by age group, 2006.**

There was little difference between hospitalisation rates for the matched cohort and for the total NHI population, but some meaningful differences between rates for the Census population and rates for the NHI population. Viewing the populations by age group (Figure 7), the absolute difference in hospitalisation rates between the Census and NHI populations remained similar until ages 65 and over, after which point the absolute difference in rate increased with increasing age. It was also at this age that a difference in rates between the matched cohort and NHI population appeared.

**Figure 8:**
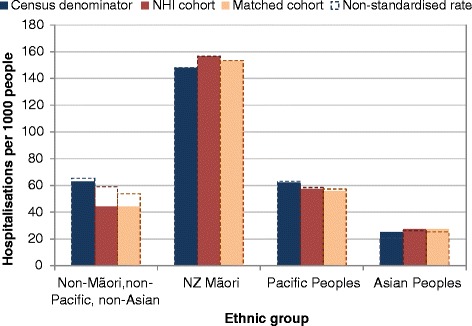
**Hospitalisation rates for 2006 Census, NHI, and matched cohort populations, by ethnic group, 2006.** *Legend:* Rates are standardised by age group, sex and NZDep2006 quintile, to the Māori population age distribution.

**Figure 9:**
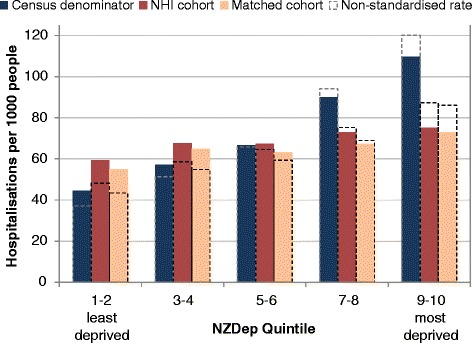
**Hospitalisation rates for 2006 Census, NHI, and matched cohort populations, by NZDep2006 quintile, 2006.** *Legend:* Rates are standardised by age group, sex and ethnic group.

Using the NHI population rather than the Census as denominator, Māori and Asian Peoples’ hospitalisation rates were marginally higher, while rates for Pacific Peoples, and Non-MPA, were lower (Figure 8). The NHI population denominator also substantially reduced the gradient of increasing hospitalisation rates with increasing socio-economic deprivation (Figure 9).

## Discussion

### NHI vs 2006 Census population comparisons

If a demographic group had a higher Census population than NHI population, it indicated that the group had less contact with health services. This could be because a) the group was not accessing health services, either because its health was good, or because its needs were not well met by health services; or b) the group included proportionally more new migrants, whose overall health was good.

If a group had a lower Census population than NHI population, likely explanations are that the group had more duplicates in the NHI, as acknowledged by the Ministry of Health; was more likely to reside outside New Zealand; or was under-counted in the Census.

#### Age distribution

The “waist” in Figure 2 reflects New Zealand’s low 1980s fertility rate. The longer waist for the NHI and matched cohort than for the Census may be a data bias. People born before 1993 were not entered into the NHI at birth, and if they remained healthy through their youth may have avoided ever being entered into the NHI.

#### Distribution by ethnic group

Differences between the NHI and Census for Asian peoples reflected a rise in immigration from Asian countries during the late 1990s and 2000s. The “healthy migrant effect” may mean that those aged 20–29 had not yet had contact with the New Zealand national health system, and were therefore not yet entered in the NHI. However, we are unsure why the Census population has a lower proportion of children aged 0–14 for Asian peoples.

In general, differences between the NHI and the Census within different age and ethnic groups should favour use of the NHI as denominator, since recorded ethnic group at hospitalisation is collected in the same context as the NHI database, and is therefore more comparable with NHI ethnic group than with Census ethnic group. However, this generality may not apply to differences as marked as those for the Asian populations.

### Hospitalisation rates

Differences in hospitalisation rates between the NHI and Census denominators for those aged over 65 mean rates for this age group should be treated with caution.

For the matched cohort, the lower hospitalisation rates in the elderly may reflect biases arising from the matching process, as elderly people are more likely than other age groups to live in multi-unit properties, which were less likely to be matched due to their numbering system.

The flattening of the socio-economic gradient in NHI-based hospitalisation rates suggests that either duplicates increased, and/or that the Census undercount increased, with socio-economic deprivation.

### NHI Data limitations

#### Duplication

The largest NHI data quality problem was the presence of duplicates: The New Zealand Ministry of Health estimated that the database included about 1 million duplicates, about 20% of the total live database. As the duplicates were unidentifiable, it was not possible to tell whether duplicates were more likely for any particular demographic groups. Some of these duplicates could be excluded by using only records updated since the NHI was introduced, but remaining numbers suggested most remained. Duplicates were also conceivably more likely to have no address data, and therefore to be excluded from the matched cohort after address matching.

Nevertheless, despite these data quality issues, comparisons of NHI age, sex, and ethnic group distribution showed it to be sufficiently close to the Census to make it a useful population.

#### Dates of birth and death

If all dates of birth and death in the NHI were correct, New Zealand would have some 3,700 people aged over 115 years (0.07% of total records). Where a date of death was recorded, additional Ministry of Health mortality data collection meant that the date was likely to be accurate. Therefore, the “overage” records were a combination of duplicates, whose deaths cannot be identified; and incorrect dates of birth. If some “overage” records had incorrect dates of birth, it is likely that other entries with more credible ages also had incorrect dates of birth.

#### Date of last update

The “date of last update” field records the most recent date the contents of any field on an individual NHI record was last changed. Where records had been updated since centralisation in mid-1993, they were more likely to represent extant persons, and the data in the record were more likely to be reliable as they were collected under the new NHI system. Therefore, we chose to include only records that had been updated.

#### Address, and fields based on address

Domicile code was based on address as collected at last contact with the health system, whether GP or hospital. If a patient did not update their GP records with a change of address, that update would not reach the NHI. The currency of address records was therefore variable, with lowest accuracy among groups with highest mobility and least contact with the health system. This population was more likely to be low-income renters as NZ has a high rate of residential mobility. Although the average tenure period was 4 years, mobility was higher among the 31%^c^ of the population who rented [20].

#### Overseas travel

The NHI database included some people who were not in New Zealand for some or all of the study period. Statistics New Zealand estimated that around 600,000 New Zealand-born, and 400,000 overseas-born New Zealand citizens lived overseas at the time of the 2006 Census^d^, in comparison to the 2006 total resident Census population of 4,027,959 people. External migration levels fluctuate from year to year, but the relevant years had the following long-term or permanent departures from New Zealand (Table 3). Permanent and long-term departures included New Zealand residents departing for an intended period of 12 months or more (or permanently), plus overseas visitors departing New Zealand after a stay of 12 months or more.

**Table 3:** **New Zealand permanent and long-term departures 2000 – 2006 (source: Statistics NZ)**

**Year**	**Permanent/long-term departures**
2000	71,045
2001	78,755
2002	59,848
2003	54,733
2004	62,277
2005	70,546
2006	69,388
**Total**	466,592

Some of the 466,592 departures would have returned before 2007; others who intended to be away less than 12 months would have stayed away longer. However, it was not possible to tell whether an NHI entry was actually in New Zealand on any given day, unless they were hospitalised.

### Generalisability

New Zealand is rare, but not unique, in its quality and quantity of nationwide health, population and other data. National health or health insurance registers exist in most of Europe and Scandinavia, and health insurance registers provide similar research potential even where they are not nationwide. This study matched to a national housing dataset, but the approach would also be useful with other address-linked health determinants, or with datasets with geographical coordinates for residences; the availability elsewhere of suitable environmental exposure datasets is outside the scope of this paper. Researchers in other countries may also face local constraints due to cultural differences in attitudes to data privacy, though this paper demonstrates that data matching can be carried out without compromising individual health information privacy.

### Recommendations

We would recommend that researchers planning to use the New Zealand NHI as a denominator population exclude records not updated since 1993, with NZDep2006 quintile unassigned, ethnic group unknown, or aged 90 and over. Lower hospitalisation rates for those aged 75 and over, and other variations by ethnic group and socio-economic deprivation, meant further adjustment of results according to NHI/Census ratios would also be adviseable. Researchers using other health and non-health-based populations could also consider using Census data to identify, exclude, and adjust for differences between study and Census populations.

## Conclusions

With appropriate exclusions and adjustments for differences from the Census, New Zealand’s NHI can be used as a denominator in public health research, and can be matched to other data sources by address to create population cohorts.

Using the NHI as a population denominator provided some advantages over the Census. First, it provided a consistent source for reported ethnic group, reducing the risk of numerator-denominator bias which can arise when basing ethnic group hospitalisation on Census counts [21,22]. Second, address linkage from the NHI allowed researchers to measure health outcomes by individual-level housing and spatial exposures. Being able to measure individual-level environmental exposures is particularly useful for public health research. Last, dates of birth and death in the NHI allowed the calculation of individual person-days of exposure.

Nonetheless, neither the NHI population nor the matched cohort were without limitations. The NHI was larger than the Census due to record duplication and entries for people residing overseas remaining on file under New Zealand addresses. NHI and QV matching was incomplete due to address data being poor quality or not suitable for matching. Both the NHI population and the matched cohort required adjustment for differences from the Census, and would be unsuitable for measuring health outcomes in people aged over 90 years or where address currency was a priority. NHI and cohort-based hospitalisation rates were higher than Census-based rates in those aged 75 and over, differed by ethnicity, and had less socio-economic gradient.

We have therefore recommended that researchers planning to use the NHI as a denominator population exclude records not updated since 1993, with NZDep2006 quintile unassigned, ethnic group unknown, or aged 90 and over; and adjust for differences in hospitalisation rates between the Census and the NHI.

### Availability of supporting data

New Zealand Census data for 2006 and other Census years are available from Statistics New Zealand. Some 2006 Census data are available online free of charge; personalised datasets are available at a small charge.

New Zealand National Health Index data and hospital admissions data are available for a small charge from the Ministry of Health, after any necessary ethical approvals have been granted.

Quotable Value New Zealand Ltd housing data, and data matching, are available at commercial rates.

### Endnotes

^a^http://www.health.govt.nz/our-work/health-identity/national-health-index/nhi-information-health-consumers/national-health-index-questions-and-answers downloaded 30 April 2009.

^b^Where the NHI data did not include a domicile code, but QV was able to match the address (see below), data from the QV Census meshblock field was used to provide a CAU instead.

^c^Sourced from Statistics New Zealand 2006 Census counts, downloaded 14 July 2014.

^d^http://www.stats.govt.nz/browse_for_stats/population/mythbusters/1million-kiwis-live-overseas.aspx

## Abbreviations

CAU: Census area unit
NHI: National Health Index
NMDS: National minimum (Hospitalisations) Dataset
Non-MPA: Non-Māori, non-Pacific, non-Asian
QV: Quotable Value New Zealand Ltd
